# National Fall Prevention Awareness Day — September 22, 2013

**Published:** 2013-09-20

Each year, one in three adults aged ≥65 years falls (1). For older persons, the consequences of falls can be devastating, including reduced mobility and loss of independence (2). Falls are responsible for >95% of hip fractures, one of the most serious fall injuries (3). In 2010, approximately 258,000 persons aged ≥65 years were hospitalized for hip fractures (4). As the U.S. population ages, the number of hip fractures is expected to increase.

However, hip fracture rates are declining among persons aged ≥65 years (5). From 1990 to 2010, annual rates decreased 20% for men, from 54.6 to 44.2 fractures per 10,000 men, and nearly 50% for women, from 125.1 to 72.3 per 10,000 women (6). These decreasing rates will partially offset the expected increase in total number of hip fractures as this age group increases.

This year, National Fall Prevention Awareness Day is September 22. As part of the campaign, older adults are encouraged to reduce their chances of falling and being injured by 1) exercising to improve their balance and leg strength; 2) having their doctor or pharmacist review their medications; 3) having their eyes checked annually by an eye doctor; 4) making home safety improvements; 5) getting adequate calcium and vitamin D; and 6) being screened for osteoporosis.

Additional information about preventing falls is available at http://www.cdc.gov/homeandrecreationalsafety/falls. CDC also provides information resources for health-care providers in its STEADI (Stopping Elderly Accidents, Deaths & Injuries) tool kit. The online resource, available at http://www.cdc.gov/injury/steadi, includes the information and tools to assess, treat, and refer older patients based on their fall risk.
